# Artificial neural network aided non-invasive grading evaluation of hepatic fibrosis by duplex ultrasonography

**DOI:** 10.1186/1472-6947-12-55

**Published:** 2012-06-20

**Authors:** Li Zhang, Qiao-ying LI, Yun-you Duan, Guo-zhen Yan, Yi-lin Yang, Rui-jing Yang

**Affiliations:** 1 Department of Ultrasound Diagnosis, Tangdu Hospital, Fourth Military Medical University, Xi'an, China

**Keywords:** Artificial neural network, Ultrasound diagnosis, Hepatic fibrosis

## Abstract

**Background:**

Artificial neural networks (ANNs) are widely studied for evaluating diseases. This paper discusses the intelligence mode of an ANN in grading the diagnosis of liver fibrosis by duplex ultrasonogaphy.

**Methods:**

239 patients who were confirmed as having liver fibrosis or cirrhosis by ultrasound guided liver biopsy were investigated in this study. We quantified ultrasonographic parameters as significant parameters using a data optimization procedure applied to an ANN. 179 patients were typed at random as the training group; 60 additional patients were consequently enrolled as the validating group. Performance of the ANN was evaluated according to accuracy, sensitivity, specificity, Youden’s index and receiver operating characteristic (ROC) analysis.

**Results:**

5 ultrasonographic parameters; i.e., the liver parenchyma, thickness of spleen, hepatic vein (HV) waveform, hepatic artery pulsatile index (HAPI) and HV damping index (HVDI), were enrolled as the input neurons in the ANN model. The sensitivity, specificity and accuracy of the ANN model for quantitative diagnosis of liver fibrosis were 95.0%, 85.0% and 88.3%, respectively. The Youden’s index (YI) was 0.80.

**Conclusions:**

The established ANN model had good sensitivity and specificity in quantitative diagnosis of hepatic fibrosis or liver cirrhosis. Our study suggests that the ANN model based on duplex ultrasound may help non-invasive grading diagnosis of liver fibrosis in clinical practice.

## Background

Hepatic fibrosis is a common feature leading to liver cirrhosis as the result of chronic hepatitis or chronic liver injury. Regardless of the causes, liver fibrosis is characterized by increased extracellular matrix forming hepatic scars [1]. Liver fibrosis is a reversible pathology process. If timely and effective treatment is adopted during the fibrosis stage, it will prevent the liver from developing hepatic cirrhosis. Liver biopsy is considered as the gold standard for final diagnosis of hepatic fibrosis, but the drawbacks, such as sampling errors, pain and complications of invasive procedures [2-4], limits its conventional use in clinical practice. Considering the need for repeated confirmation of the condition of the liver during treatment, a non-invasive modality for grading liver fibrosis or cirrhosis is urgently needed. Imaging technology has an advantage in being noninvasive and allowing repeated maneuverability. Among other methods, ultrasound scanning is the most frequently used with the superiority of inexpensive, real-time imaging and hemodynamic evaluation ability. Many researchers have been committed to establishing a system for liver fibrosis diagnosis or fibrotic stage evaluation by ultrasound [5,6].

In recent years, artificial neural networks (ANNs) have appeared as tools for clinical decision-making [7] and are potentially more successful than traditional statistical models in predicting clinical outcomes [8,9]. ANNs can learn experiential knowledge expressed through internal connections in a similar way to how natural neurons function in the brain and this knowledge can be made available for use [10]. According to the lesion characteristics of liver fibrosis or cirrhosis, one single ultrasonographic index is incapable of reflecting the whole problem. An ANN is suitable for a “multi-parameter” diagnosing mode. In previous studies, liver fibrosis or cirrhosis was graded by an ANN based on laboratory results [11,12]. The results showed that a three-layer ANN could effectively identify the risk for liver fibrosis in chronic hepatitis B (CHB) patients with positive HBsAg. An ANN consisting of an input layer, an output layer and one or more hidden layer could be adequate as a universal approximator of any nonlinear function [11,13]. The input layer comprises the data available for analyzing and the output layer comprises the outcome such as diagnosis, prognosis and evaluation.

In this study, we established an ANN model based on the ultrasonographic changes in patients with liver fibrosis for the first time and aimed to predict the risk of early liver cirrhosis. We also assessed the early warning ability of the ANN from an ultrasonographic angle.

## Methods

### Ethical approval of the study protocol

All subjects included in the study provided written informed consent. The study protocol was approved by the ethics committee of the Fourth Military Medical University Tangdu Hospital (Xi’an, China).

### Patient selection

Between July 2008 to June 2010, 308 patients infected by hepatitis B virus (HBV) or hepatitis C virus (HCV) and confirmed by laboratory tests at Tangdu Hospital of Fourth Military Medical University, were investigated in this study. The pathological changes in these patients to fibrosis or cirrhosis were evaluated by ultrasound-guided liver biopsy. Finally, 239 patients with liver pathological changes were enrolled in the study. Of these patients, 170 were affected by HBV and 69 by chronic HCV. Subjects with hematonosis and heart disease were excluded from the study. The patients were randomly divided into a training group (179 cases; 75%) and a validation group (60 cases; 25%).

### Liver pathologic evaluation

Ultrasound-guided liver biopsy was performed after ultrasound examination. Three pathologists that had no clinical information about the patients evaluated the degree of hepatic fibrosis. Fibrosis was staged from F0 to F4 according to the METAVIR system: F0, no fibrosis; F1, portal fibrosis without septa; F2, few septa; F3, numerous septa without cirrhosis; and F4, cirrhosis [12]. In the present study, fibrosis was defined as F1 to F3 stages; cirrhosis was considered as F4 stage.

### Ultrasound examination

Duplex ultrasonographic examinations were conducted with the Acuson Sequrie 512 (Siemens Acuson Co., Mountain View, California) using a 3.5 MHz phased array transducer and a 10 MHz high-resolution transducer. All studied subjects fasted overnight before the examination. Grey-scale parameters including the liver parenchyma, liver envelope, the size of the spleen, ascite and Doppler parameters of intrahepatic blood vascular such as hepatic artery pulsatile index (HAPI), portal vein velocity (PVVel), hepatic vein damping index (HVDI) were assessed. The liver parenchyma and liver envelope were observed by a high-resolution transducer. Four variables such as the liver parenchyma, liver envelope, ascite and hepatic vein waveform were graded from 0 to 2 according to the severity of pathological changes, as shown in Table 1. Measurements of each parameter were made during suspended respiration in the same area.

**Table 1:** Grading standard for the evaluation of ultrasonographic changes on liver fibrosis

** *Variables* **	**0**	**1**	**2**
Liver envelope	Smooth	slightly coarse	obviously coarse or like wavy changes
Liver parenchyma	Homogeneous	Heterogenous	coarsened
Ascites	no or < 500 ml	500 ml ~ 3000 ml	> 3000 ml
HV waveform	Triphasic	Biphasic	monophasic

The Doppler gate was placed in the porta hepatis to measure the relevant parameters of the portal vein and hepatic artery. Velocity measurements were conducted at 30– 60°. The mean velocity of PVVel and HAPI were calculated automatically by the machine after the waveform trace for three cardiac cycles were obtained. Doppler hepatic vein (HV) waveforms were recorded for at least 5 s with end-expiration breath holding. The middle HV was measured because it has the most consistent triphasic flow in healthy subjects and the most favorable Doppler angle. The Doppler gate was placed in the vessel 2–3 cm away from the inferior vena cava (IVC) to measure the HV waveform. HV waveforms were classified as ‘triphasic’ (reversed flow in at least one phase), ‘biphasic’ (no reversed flow with or without decreased phasic oscillation), or ‘monophasic’ (flat with or without fluttering). Two examiners (Yilin Yang and Guozhen Yan) classified the recorded HV Doppler waveform tracings. The damping index (DI) was calculated by the minimum velocity/maximum velocity of downward HV flow.

Doppler examinations were undertaken by one author (Li Zhang) without prior knowledge of the clinical and biochemical status of the study population. The reproducibility of this method was evaluated with repeated ultrasound measurements of portal venous blood flow velocity in 10 healthy subjects over 5 consecutive days [14]. In order to minimize inter-observer variation, at the beginning we unified the method of measuring each index, and all parameters were measured by the same observer who had no knowledge of the patients’ conditions on the same machine. Each index was calculated as the mean of three consistent measurements. The Doppler parameters we measured were consistent in all subjects.

### Development of an ANN model

ANN models were constructed by using neural-network software (Statistic Neural Networks, version 4.0). The architecture of the ANN consisted of three layers; i.e., the input, hidden and output layers. Each layer contained 5 neurons, 11 neurons and 1 neuron, respectively. Neurons were tied together with weighted connections. The number of the network layers, hidden neurons and the stopping criteria were determined through a trial-and-error process. The input layer simply fed information, as well as related predictive factors, into the network, while nodes in the hidden and output layers processed information. The input data selected for the development of the neural network were ultrasongraphic parameters. The output layers contained one neuron (0, fibrosis; 1, cirrhosis).

The training rule that was used was back-propagation of error. During the training, the corresponding known outputs of the system were held in the output nodes to compare with the results produced by the network. The nodes in the hidden layer had no prescribed initial values and helped to allow complex relationships between the input and output nodes to evolve. Information was transported from the input layer to the output layer by calculating the sum at each node, which was derived from combining all the nodes in the previous layer. Training was terminated when the sum of square errors was at a minimum. At the end of each training session, the network was tested and the prediction accuracy was calculated. We then selected the best network in terms of accuracy.

### Statistical analysis

Continuous variables were expressed as mean ± standard deviation (SD). Categorical variables were compared using χ^2^ analysis and continuous variables were compared by the Mann–Whitney U Test, or Kruskal-Wallis Test. Performance of the ANN prediction was tested using receiver operating characteristic (ROC) curve analysis. The ANN predictions for the diagnosis of liver fibrosis stage were expressed in terms of accuracy, sensitivity, specificity, positive predictive value (PPV), negative predictive value (NPV) and Youden’s index (YI) for several considered cut-off values. A value of *p*<0.05 was considered significant in all the analyses.

## Results

Of the initial 308 subjects, 69 patients, who were confirmed to be without fibrosis were excluded from the study. The fibrosis group contained 157 subjects, 53(33.7%) had portal fibrosis without septa (F1), 30 (19.1%) had few septa (F2), and 74 (47.1%) had numerous septa without cirrhosis (F3); the cirrhosis group contained 82 subjects, respectively. The main clinical and pathological data for the patients according to the fibrosis stage and study group at the beginning are presented in Table 2. We randomly divided the 239 patients who underwent liver biopsy into 2 groups: a training group and a validating group.

**Table 2:** The distribution and clinical characteristics of 239 subjects

	** *Fibrosis (F1-F3)* **	** *Cirrhosis (F4)* **
Age (median/range)	45(30–63)	47(26–59)
Gender (F/M)	62/117	15/45
Post-hepatitis B	128	42
Post-hepatitis C	51	18
Training group	146	33
Validating group	33	27
	** *Training Group* **	** *Validating Group* **
Age (median/range)	43(35–57)	45(26–63)
Gender (F/M)	57/121	20/51
Post-hepatitis B	120	50
Post-hepatitis C	62	7
** *Stage of Liver Fibrosis* **		
F1	40	13
F2	22	8
F3	55	19
F4	62	20

After statistical analysis, 5 ultrasonographic variables; i.e., the liver parenchymal, thickness of the spleen, the HV waveform, HAPI and DI, were found to be significantly different between the fibrosis group and cirrhosis group, and were subsequently selected as the input neurons (Table 3).

**Table 3:** Statistical comparison of the ultrasonographic viriables between the fibrosis group and the cirrhosis group

** *Variable* **	** *Fibrosis group* **	** *Cirrhosis group* **	** *P value* **
Live parenchymal	0.645 ± 0.055	0.816 ± 0.129	0.022*
Liver envelope	0.639 ± 0.054	0.709 ± 0.112	0.224
Thickness of Spleen (cm)	3.279 ± 0.439	4.058 ± 0.672	0.003*
Ascites	0.413 ± 0.035	0.516 ± 0.082	0.976
HV waveform(0/ι/П) ^a^	128/8/3	28/6/6	<0.0001*
PVVel (cm/s)	18.16(1.273)	15.827(6.301)	0.114
HAPI	1.247 ± 0.155	1.147 ± 0.283	0.009*
HARI	0.697 ± 0.050	0.711 ± 0.052	0.910
DI	0.458 ± 0.131	0.574 ± 0.111	0.030*

* Significant differences (P < 0.05).

^a^ Absolute number.

**Figure 1:**
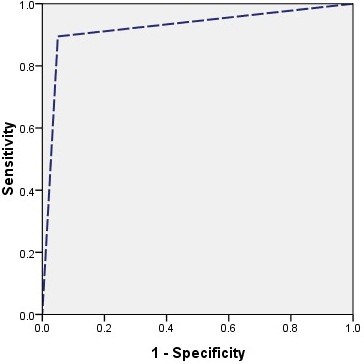
ROC (receiver operating characteristic) curves of ANN (artificial neural network).

Table 4 shows the ANN performance in diagnosing cirrhosis in chronic HBV patients, compared to the gold standard liver biopsy. Some predictive performance indices such as sensitivity, specificity, misdiagnosis rate (MR), PPV (positive predictive value), NPV (negative predictive value), accuracy, YI (Youden’s index) and AUC are listed in Table 5. ROC curves for the ANN model are shown in Figure 1.

**Table 4:** Ultrasound diagnosis of neural network used in the results of liver fibrosis

	** *Pathology* **		
** *ANN* **	** *Fibrosis (F1-F3)* **	** *Cirrhosis(F4)* **	** *Total* **
** *Fibrosis (F1-F3)* **	38	3	41
** *Cirrhosis(F4)* **	2	17	19
** *Total* **	40	20	60

**Table 5:** Predictive performance of ANN (artificial neural network)

	** *sensitivity* **	** *specificity* **	** *MR* **	** *PPV* **	** *NPV* **	** *Accuracy* **	** *YI* **	** *AUC* **
**ANN**	95.0 %	85.0 %	8.3 %	92.6 %	89.4 %	88.3 %	0.80	0.922

## Discussion

Currently, with the development of laboratory and imaging means for staging the fibrotic evolution of chronic liver diseases, clinical validation has highlighted the fact that, overall, liver biopsy is probably an imperfect gold standard [15]. Actually, even a 25 mm long liver biopsy has a 25% rate of discordance for fibrosis staging [16]. Therefore, liver biopsy is prone to sampling errors and to intraobserver and interobserver variability [17,18]. Also, when the specimen size is adequate, the level of experience of the pathologist may even be more important [19]. Invasive procedures are not suitable for regular clinical monitoring of disease progression. Even though there is a high prevalence of chronic liver disease worldwide and it represents a significant public health problem, liver biopsy is obviously not appropriate for screening liver fibrosis and cirrhosis.

Liver fibrosis is a kind of diffuse lesion involved in multiple structures of the liver. Several factors are taken into account, either by imaging or laboratory tests, before the diagnosis of liver fibrosis or cirrhosis can be made. When multiple and diverse factors are likely to influence decision making, computer-based decision support systems, such as neural networks, are capable of handling large amounts of data and are helpful in arriving at or supplementing a correct decision by clinicians [20,21]. ANNs have been used in medicine for various purposes, including prediction of mortality of patients with cirrhosis of the liver [22,23]. An intelligent mode was also compared to MELD scoring, Child–Pugh’s scoring and other conventional logistic regression models and performance of an ANN was significantly better than those of the models.

Real time ultrasonography has become an integral part of the non-invasive evaluation of chronic liver disease in many clinical settings while the search for a non-invasive imaging marker for staging liver fibrosis or cirrhosis is inactive. The performance of ultrasonographic imaging as a non-invasive diagnostic or prognostic modality for liver fibrosis or cirrhosis, as well as for correlation with histological changes and functional disorders of the liver, remains controversial and is still debated. However, recent advances in ultrasound technology have improved the diagnostic accuracy of fibrosis in patients with chronic liver disease. Aube et al. [24] studied a high-resolution ultrasound probe of the liver parenchyma, liver surface smoothness, spleen size and portal vein blood flow rate, using 11 indicators in ultrasonic testing, and found an accuracy of 82 ~ 88% for surface nodular changes in the liver and spleen thickness in the diagnosis of cirrhosis of the liver.

In the present study, we constructed a multi-parameter dependent diagnostic model based on ultrasound in order to avoid the shortcomings of a single-parameter decision making model of ultrasonography. We took several ultrasonographic variables into consideration including grey-scale and Doppler indexes such as the liver parenchyma, liver edge, PVVel, and HAPI. Secondly, according to the different treatment principles for liver fibrosis and liver cirrhosis, we divided the patients into two groups: the fibrotic group (F1-F3 stage) and the cirrhotic group (F4 stage). Variables like the liver parenchyma, liver envelope, ascites and HV waveform were graded from 0 to 2 according to the imaging changes in different stages. The variables were quantitatively described, for example, as PVVel, HAPI, HARI and DI, and were compared by the actual values. DI was used to quantitatively assess the extent of the abnormal HV waveform. The relatively large number of intra- and extra-hepatic variables was considered in the study to work with the largest possible amount of information. In fact, data collection was performed by trying to include all variables that could have a connection with the problem. However, some of these variables may contain confusing information, or even completely irrelevant information. Selecting the significant variables after statistical analysis can increase diagnostic accuracy as well as sensitivity and specificity. Some experts would consider non-invasive serum tests of fibrosis with AUC-ROC values of 0.85 to 0.90 to be as good as a liver biopsy for staging fibrosis [25]. In our study, the diagnostic performance achieved by the ultrasound-based ANN was considered as having AUC-ROC values around 0.92.

### Limitation of the study

This study has several limitations that must be taken into account. Firstly, the ultrasound variables in the present study did not fully cover all involved parameters, although some of these variables could have contributed to improvement of the ANN. The ANN model was constructed using 10 variables as the proposed input neurons. This Secondly, the number of patients was limiting. In an ANN model, each group should have 100 patients to avoid the risk of overfitting the data. This was not fully achieved for the validation group (60 of 239 patients). Finally, we could not evaluate the accuracy of pathological diagnosis caused by sampling error or variation in the experience of the pathologists.

## Conclusions

The study highlighted the construction and assessment of an ANN for identifying the risk of liver cirrhosis by a non-invasive imaging modality. In the study, we provided evidence that this intelligent model can accurately predict liver cirrhosis by ultrasound. It could be used to improve clinical decisions for patients with chronic liver disease.

## Competing interests

The authors declare that they have no competing interests.

## Authors’ contributions

LZ performed the ultrasound examinations and imaging analysis. QYL performed the data analysis. YLY and GZY participated in the ultrasound imaging analysis. YLY and RJY performed the liver biopsy under the guidance of ultrasound. YYD edited the article. All authors read and approved the final manuscript.

## Pre-publication history

The pre-publication history for this paper can be accessed here:

http://www.biomedcentral.com/1472-6947/12/55/prepub
